# The High Prevalence of Osteoporosis as a Preventable Disease: The Need for Greater Attention to Prevention Programs in Iran

**Published:** 2018-08

**Authors:** Amin DOOSTI-IRANI, Mahin GHAFARI, Zahra CHERAGHI

**Affiliations:** 1. Dept. of Epidemiology, School of Public Health, Hamadan University of Medical Sciences, Hamadan, Iran; 2. Modeling of Noncommunicable Diseases Research Center, School of Public Health, Hamadan University of Medical Sciences, Hamadan, Iran; 3. Dept. of Public Health, School of Health, Shahrekord University of Medical Sciences, Shahrekord, Iran

## Dear Editor-in-Chief

Osteoporosis is a skeletal system disease diagnosed following a reduction in bone mass density. Osteoporosis is an important cause of bone fractures. It can also increase hospitalizations due to secondary complications and is an important threat to the life of older adults (1). According to the latest statistics published in Iran, the overall prevalence of osteoporosis is 17%. This prevalence was reported as 12% in men and as 19% in post-menopausal women. Approximately 35% of the adult and older adult population of the country is affected by osteopenia (2). Furthermore, about two million people are at risk for osteoporotic fractures in Iran (3).

Osteoporosis is an important problem among the older adult population, and this issue is further exacerbated by the higher life expectancy and consequently the increased older adult population in Iran. According to WHO, life expectancy was 74.77 yr in 2015 (4), 71.9 yr for men and 78 yr for women (5). This index is indicative of population aging in Iran. The prevalence of osteoporosis as a prevalent disease among older adults is expected to further increase in Iran in the future and may become a detrimental threat to the health and life of Iranian older adults.

The direct costs of osteoporotic hip fractures were 28 million USD in 2010 in Iran and these costs are expected to reach 51 and 250 million USD by 2020 and 2050 (3). Osteoporosis is a largely preventable disease associated with major controllable risk factors such as poor nutrition, smoking, low physical activity and the abuse of corticosteroids (1). The consumption of milk and dairy products as an important factor in helping prevent osteoporosis is half its global standard in Iran. The annual per capita consumption of dairy should be about 165 kg; however, this figure is approximately 85 kg in Iran (6). Moreover, the prevalence of vitamin D deficiency as another risk factor for osteoporosis was reported as 10% to 50% in Iran (7–9).

Given the existing evidence on osteoporosis and its risk factors in Iran and the phenomenon of population aging in the country, osteoporosis is expected to affect a large proportion of the Iranian population in the years to come. The facts about the disease in Iran can be an alarm for health policymakers. Planning for primary preventive actions for osteoporosis is therefore essential for improving the health and quality of life of the Iranian population, especially its older adult population.

## References

[1] Cosman F de Beur SJ LeBoff MS (2014). Clinician’s Guide to Prevention and Treatment of Osteoporosis. Osteoporos Int, 25:2359–81. 25182228 10.1007/s00198-014-2794-2 PMC4176573

[2] Doosti Irani A Poorolajal J Khalilian A (2013). Prevalence of osteoporosis in Iran: A meta-analysis. J Res Med Sci, 18:759–66. 24381618 PMC3872583

[3] El-Hajj Fuleihan G Adib G Nauroy L (2011). The middle east & Africa regional audit, epidemiology, costs & burden of osteoporosis in 2011. International Osteoporosis Foundation, Switzerland pp. 102011–105000.

[4] WHO Iran (Islamic Republic of): WHO statistical profile. http://www.who.int/countries/irn/en/

[5] Institute for Health Metrics and Evaluation Iran: How long do people live?. http://www.healthdata.org/iran.

[6] IRNA Iran’s per capita milk consumption. http://www.irna.ir/en/News/2716240/Social/Iran%E2%80%99s_per_capita_milk_consumption

[7] Hashemipour S Larijani B Adibi H (2004). Vitamin D deficiency and causative factors in the population of Tehran. BMC Public Health, 4:38. 15327695 10.1186/1471-2458-4-38 PMC517720

[8] Hovsepian S Amini M Aminorroaya A (2011). Prevalence of vitamin D deficiency among adult population of Isfahan City, Iran. J Health Popul Nutr, 29:149–55. 21608424 10.3329/jhpn.v29i2.7857 PMC3126987

[9] Moussavi M Heidarpour R Aminorroaya A (2005). Prevalence of vitamin D deficiency in Isfahani high school students in 2004. Horm Res, 64:144–8. 16192739 10.1159/000088588

